# Inductive Sensor Characteristics for Conductivity Measurement of Non-Ferromagnetic Metals Based on Single-Layer Solenoid

**DOI:** 10.3390/s25175566

**Published:** 2025-09-06

**Authors:** Huan Wang, Ziyi Han, Yongjian Chen, Shuyu Li, Haoran Li, Hao Shen, Chunlong Xu

**Affiliations:** 1 School of Science, Chang’an University, Xi’an 710054, China; shenhao@chd.edu.cn; 2 School of Future Transportation, Chang’an University, Xi’an 710054, China; 2022901164@chd.edu.cn (Z.H.); 2022902106@chd.edu.cn (Y.C.); 2022905123@chd.edu.cn (S.L.); 2022905121@chd.edu.cn (H.L.)

**Keywords:** inductive sensor, eddy current, finite-element method, electric conductivity, RLC resonant circuit

## Abstract

For the measurement of electrical conductivity of metal materials, the traditional contact measurement method has a limited test range and requires periodic electronic calibration. In order to overcome the above shortcomings, this paper takes the inductive response of an RLC circuit driven by alternating sources as the research object and proposes a non-contact method for conductivity measurement of non-ferromagnetic metals engaged by a single-layer solenoid sensor. The effect of the circuit parameters on the inductive sensor characteristics has been described with different resonant modes, and the electric conductivities of different metals can be theoretically calculated based on eddy current. Moreover, the Comsol Multiphysics software is used to conduct finite element analysis to compare the experimental results and the simulation, which is consistent with the theoretical analysis. The measured accuracy of the inductive sensor is verified to be higher than 91% in parallel resonance, which exhibits higher stability and precision than that of series mode. The implementation of this project will provide the theoretical basis and data reference for the detection of electromagnetic properties of unknown metals and has a wide range of applications in non-destructive testing, engineering construction detection, and other fields.

## 1. Introduction

Benefiting from the simple structure and high reliability, the non-contact eddy current testing (ECT) has been extensively utilized in the measurement of electromagnetic properties of metals [1], the detection of material defects [2], the clearance testing [3], imaging [4], the thickness measurement [5], and so on. The traditional method for electric feature measurement, such as the four-point probe method [6,7], suffers from complex structural design and requires periodic electronic calibration. In order to satisfy the high performance of the detection, inductive sensors with low cost, high stability, and high precision are desired. The past decades have witnessed a rapid development of the non-contact ECT technologies [8,9]. As a typical non-contact method, coil-based inductive sensors can take advantage of non-destructive examination (NDE) with high efficiency. Meanwhile, it is adaptive to measure the electric properties of the metal materials, which play a crucial role in the circuit fabrication and power transportation.

Among the coil-based inductive sensors for electrical testing, traditional conductivity measurement is engaged by the transform-type sensor, consisting of the excitation coil and pick-up coil [10]. To activate the eddy current, the time-varying magnetic field is produced when the alternating current passes through the coil. Based on Faraday’s law, the induced electromagnetic force (Emf) can be excited by the variation in the magnetic flux, and it drives the electron to move and further form the eddy current, which produces the difference in the inductance [11] and impedance [12] of the surrounding coil. Henceforth, many studies have been conducted to clarify the relationship between the measured data and the conductivity, which has developed from single-parameter to parameter-fusion sensing [13,14]. The modeling description of the inductive conductivity sensor, which is derived from the Maxwell equation, has also been proposed [15]. Since the inductance variation tuned by inserting metals can be transformed into the voltage output of the solenoid inductor, the resonant inductive circuit has also been applied in the conductivity sensor to further promote the accuracy, which is implemented by measuring the inductance change in the coil [16]. Nevertheless, the above-mentioned measurement is applied based on a double-coil configuration, which leads to the complexity of the testing system and high cost.

Unlike the double-coil structure, the single-coil sensor based on the sensitive elements with a simple symmetrical shape can serve not only as an excitation coil to generate an electromagnetic field but also as a receiving coil to read the measurement signal. The conductivity measurement implemented by a single-coil sensor can simplify the experimental setup with low cost. Due to this structure lacking strong coupling characteristics in eddy currents [17], the self-inductance of the single-coil inductive sensor is relatively low, which accounts for the weak influence of the inductance change induced by metals. However, with the application of the resonant technology, the difference in inductance caused by eddy current can be enhanced significantly, which enables the single-coil structure to have the possibility of measuring low-conductivity media. Moreover, the single-coil configuration possesses lower volume and weight, which helps to conduct the measurement in tough and flexible circumstances, such as seawater [18], lubrication oil [19], and carbon fiber composite [20]. For example, Wang et al. have reported the conductivity measurement of a liquid-solid mixture based on a single-layer coil, demonstrating that the prediction errors of the conductivity are less than 5.0% [21]. To further verify the reliability and accuracy, the single-coil sensor operated in high temperature has been carried out to identify the impact caused by external factors [22]. By combining the inductive and capacitive response, the insulator-conductor hybrid structure has been pointed out to perform the NDE by using the single spiral coil sensor [23]. In addition, the single-coil sensor can be employed to implement the real-time electromagnetic induction imaging, which is regarded as an effective approach for obtaining more diverse information [24]. These works have proved that the single-coil inductive sensor has extensive electromagnetic application aspects. However, the influence factors of single-coil inductive sensors are mainly focused on the species of the objectors, coil shape, and sensor placement on the measured object [25,26,27], while the effect of different resonant modes and circuit parameters on the conductivity measurement has still not been systematically investigated. Therefore, it is highly desired to study the resonant characteristics based on the single-coil system, which has significance for the comprehensive understanding of the inductive sensor properties and the confirmation of the application scope for conductivity measurement.

Herein, we construct an effective model of a single-layer solenoid inductor for conductivity measurement based on the RLC resonant circuit by analyzing the inductance variation in different circuit parameters induced by the eddy current effect. The inductive sensor characteristics under different resonant modes have been investigated, and the feasibility of the single-layer solenoid sensor for the electrical conductivity measurement is verified by both experimental and theoretical methods. The inductance change tuned by inserting non-ferromagnetic metals with different radii is derived from the alternating voltage output of the solenoid inductor. Meanwhile, the comparison of series and parallel resonance on measured conductivity is observed, indicating that the measurement engaged in parallel mode is more stable than that of series mode. It also turns out that the measured conductivity possesses high accuracies, which fit better with the known values of conductivity in the literature. Furthermore, simulation results also confirm the mechanism of the inductive sensor, which is executed by the finite-element method (FEM). Our experiments are expected to supply a theoretical basis for the single-solenoid sensor in non-contact conductivity measurement and can be useful in various engineering applications.

## 2. Inductive Sensor Model and Measurement Method

### 2.1. Inductive Model of the Single-Layer Solenoid with a Finite Length

When an external metal passes through a single-layer solenoid, it excites an additional magnetic field that is generated, which superimposes onto the original field of the coil. This alteration in the total magnetic field surrounding the coil can induce a change in its inductance. In the following, the model description for the relation between the inductance and the electric conductivity can be derived from Maxwell’s electromagnetic theory.

**Figure 1:**
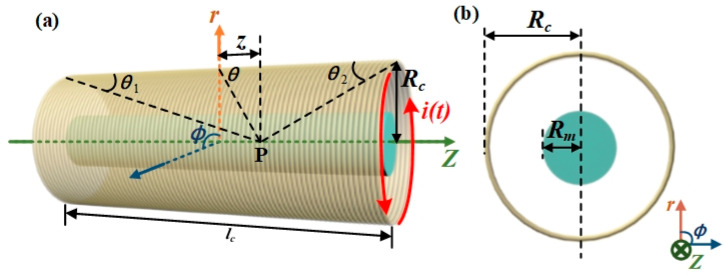
(**a**) Schematic illustration of the solenoid inductor filled with the testing metal. (**b**) The cross-sectional view of the solenoid inductor.

The inductive model of the metal-cored solenoid sensor can be described by Maxwell’s equation, which is induced by the eddy current. The inductance of the pure (or air-cored) solenoid is denoted by *L*_0_, while *L* refers to the inductance of the solenoid inserted with the testing metal (with conductivity of *σ_m_*). Since the analytical solution of the coil inductance change can be acquired only when the field distribution is symmetrical, it is assumed that the metal shape is cylindrical in our experiment. The geometric parameters of the inductive sensor are depicted in Figure 1. The solenoid with the initial turn of the coil (*N*) of 480, the radius (*R_c_*) of 12 mm, and the length (*l_c_*) of 60 mm is set in this configuration. The radius and the length of the metal rod are represented by *R_m_* and *l_m_*, respectively.

By considering the cylindrical coordinate system (*r*, *ϕ*, *Z*), where the *Z*-axis is along the axis of the solenoid, the magnetic induction ***B*** is axisymmetric and varies along the *Z*-direction. According to Biot-Savart law [28], the magnitude of ***B*** for a single coil is given by:

(1) $$B=\frac{\mu_{0}R_{c}^{2}i\left(t\right)}{2{\left(R_{c}^{2}+z^{2}\right)}^{3/2}}$$

where the horizontal coordinate of point *P* (any point on the center axis of the coil) is *z*. The alternating current of the coil is denoted as *i*(*t*), and *μ*_0_ represents the permeability of vacuum.

As shown in Figure 1a, it is supposed that *n* is the number of turns per unit length of the solenoid defined by *N*/*l*_c_, and d*l* is the per unit length of the coil. The magnetic induction caused by *ndl* turns coil is given by:

(2) $$dB=\frac{\mu_{0}R_{c}^{2}i\left(t\right)}{2{\left(R_{c}^{2}+z^{2}\right)}^{3/2}}ndl$$

Thus, the magnetic induction at the point *P* induced by the air-cored solenoid can be expressed as:

(3) $$B=\frac{\mu_{0}ni\left(t\right)}{2}\overset{\frac{l_{c}}{2}}{\underset{-\frac{l_{c}}{2}}{\int }}\frac{R_{c}^{2}dl}{{\left(R_{c}^{2}+z^{2}\right)}^{3/2}}=\frac{\mu_{0}ni\left(t\right)}{2}\overset{\frac{l_{c}}{2}}{\underset{-\frac{l_{c}}{2}}{\int }}\frac{\left.R_{c}^{2}d(R_{c}\cot\theta \right)}{{\left(R_{c}^{2}\;/\;\sin^{2}\theta \right)}^{3/2}}=\frac{\mu_{0}}{2}ni\left(\cos\theta_{1}-\cos\theta_{2}\right)$$

where *θ*_1_ and *θ*_2_ are the angles when the angle *θ* is at the two ends of the solenoid, as shown in Figure 1.

To promote the accuracy of testing conductivity, we need to consider the edge effect of the magnetic induction. Suppose that the magnetic induction inside the air-cored solenoid is uniform; the average value of the magnetic induction can be deduced as:

(4) $$\begin{array}{ll} B_{P} & =\frac{1}{l_{c}}\overset{\frac{l_{c}}{2}}{\underset{-\frac{l_{c}}{2}}{\int }}Bdz=\frac{\mu_{0}}{2l_{c}}ni\overset{\frac{l_{c}}{2}}{\underset{-\frac{l_{c}}{2}}{\int }}(\cos\theta_{1}-\cos\theta_{2})dz\\ & =\frac{\mu_{0}}{2l_{c}}ni\overset{\frac{l_{c}}{2}}{\underset{-\frac{l_{c}}{2}}{\int }}\left[\frac{z+\frac{l_{c}}{2}}{\sqrt{R_{c}^{2}+{\left(z+\frac{l_{c}}{2}\right)}^{2}}}-\frac{z-\frac{l_{c}}{2}}{\sqrt{R_{c}^{2}+{\left(z-\frac{l_{c}}{2}\right)}^{2}}}\right]dz\;\;\;(let\;u=z+\frac{l_{c}}{2},\;so\;du=dz)\\ & =\frac{\mu_{0}}{2l_{c}}ni\overset{l_{c}}{\underset{0}{\int }}\left[\frac{u}{\sqrt{R_{c}^{2}+u^{2}}}+\frac{l_{c}-u}{\sqrt{R_{c}^{2}+{\left(l_{c}-u\right)}^{2}}}\right]du\;\;\;\;\;\;\;\;\;\;\;\;\;\;\;\;\;(let\;u^{\prime }=l_{c}-u_{\prime }\;so\;du^{\prime }=-du)\\ & =\frac{\mu_{0}}{2l_{c}}ni\left[\overset{l_{c}}{\underset{0}{\int }}\frac{u}{\sqrt{R_{c}^{2}+u^{2}}}du+\overset{0}{\underset{l_{c}}{\int }}\frac{u^{\prime }}{\sqrt{R_{c}^{2}+u^{\prime 2}}}(-du^{\prime })\right]\\ & =\frac{\mu_{0}}{2l_{c}}ni\left[\left(\sqrt{R_{c}^{2}+l_{c}^{2}}-R_{c}\right)+\left(\sqrt{R_{c}^{2}+l_{c}^{2}}-R_{c}\right)\right]\\ & =\frac{\mu_{0}Ni}{l_{c}^{2}}\left(\sqrt{R_{c}^{2}+l_{c}^{2}}-R_{c}\right) \end{array}$$

The inductance of the air-cored solenoid is obtained as:

(5) $$L_{0}=\frac{NB_{P}\pi R_{c}^{2}}{i\left(t\right)}=\frac{\mu_{0}N^{2}\pi R_{c}^{2}}{l_{c}^{2}}\left(\sqrt{R_{c}^{2}+l_{c}^{2}}-R_{c}\right)$$

This inductive model can contribute to a more precise change in inductance, which helps to promote the accuracy of the conductivity measurement. Specifically, by considering the condition that the length of the solenoid (*l_c_*) is much larger than the coil radius (*R_c_*), the inductance of the solenoid can be simplified as: $L_{0}=\mu_{0}n^{2}\pi R_{c}^{2}l_{c}$.

### 2.2. Equivalent Model of Electrical Conductivity

As for the measurement of electric conductivity, the characteristics of ferromagnetic metals vary from non-ferromagnetic metals. Due to the magnetic permeability of the ferromagnetic metal being pretty large, the magnetization effect is greater than the eddy current effect, which contributes to a positive increment for the inductance. More importantly, the hysteretic relation between the magnetic induction ***B*** and the magnetic field ***H*** cannot be ignored, which will make the electromagnetic response of ferromagnetic metals more complex. However, the relative permeability of the non-ferromagnetic metal is approximately 1, indicating that there is no magnetization. This result means that the relation between ***B*** and ***H*** has a linear dependency, denoted as ***B*** = *μ*_0_*μ*_r_***H***. Hence, an additional magnetic field can only be stimulated due to the effect of eddy current, which leads to a negative change for the inductance [28]. To obtain a more precise analytic solution of conductivity, we mainly focus on the conductivity model of non-ferromagnetic metals.

According to Faraday’s law [15] and Maxwell’s equations, the induced electromotive force is denoted as:

(6) $$\varepsilon \left(t\right)=-\frac{d\phi }{dt}$$

(7) $$\nabla \times E=-\frac{\partial B}{\partial t}$$

where the *ε*(*t*) denotes the induced electromotive force as a function of time *t*, *ϕ* represents the magnetic flux, and ***E*** marks the induced electric field.

Suppose that the metal cylinder is located inside a long straight solenoid, and it carries a time-varying current *i*(*t*). Based on Equation (4), the average magnetic induction *B*(*t*) of the single-layer solenoid is regarded as a uniform axial magnetic field (by considering the edge effects) [29], whose magnitude can be expressed as:

(8) $$B\left(t\right)=\mu_{0}n^{\prime }i\left(t\right)\;\;\;\;\;\;\;\left(n^{\prime }=\frac{N}{l_{c}^{2}}\left(\sqrt{R_{c}^{2}+{l_{c}}^{2}}-R_{c}\right)\right)$$

According to Maxwell’s equation, the induced electric field (***E***) has only the circumferential component *E_ϕ_* as a function of radial distance *r*. Therefore, the differential form of Maxwell’s equation can be simplified as:

(9) $$\nabla \times E_{z}=-\frac{1}{r}\frac{\partial }{\partial r}\;\left(rE_{\varphi }\right)=-\frac{\partial B_{z}}{\partial t}$$

(10) $$E_{\varphi }=\frac{1}{2}r\mu_{0}n^{\prime }\frac{di}{dt}$$

Subsequently, the induced eddy current *J*_φ_ (*r*,*t*) in the metal rod is defined by Ohm’s Law:

(11) $$J_{\varphi }\left(r,t\right)=\sigma E_{\varphi }\left(r,t\right)=\sigma_{m}\frac{1}{2}r\mu_{0}n\prime \frac{di}{dt}$$

where *σ_m_* denotes the conductivity of the metal.

It is known that the amplitude of the eddy current can be significantly influenced by the non-contact lift-off variation (the distance between the sensing probe and the testing metal) [30]. To avoid the disturbance induced by the lift-off effect, all the parameters are measured when the metal rod is completely inserted into the solenoid. By neglecting the displacement current [15] and applying Ampere’s circular law, for *r* < *R_c_*, the change in the magnetic induction *B’* and the magnetic flux caused by the eddy current (*Φ_σ_*) are given by:

(12) $$B^{\prime }\left(r\right)=\mu_{0}\overset{R_{m}}{\underset{r}{\int }}J_{\varphi }dr=\frac{1}{4}\mu_{0}^{2}n^{\prime }\sigma_{m}\frac{di}{dt}\left({R_{m}}^{2}-r^{2}\right)$$

(13) $$\Phi_{\sigma }=N\overset{R_{m}}{\underset{0}{\int }}B^{\prime }\left(r\right)2\pi rdr=nl\overset{R_{m}}{\underset{0}{\int }}B^{\prime }\left(r\right)2\pi rdr=\frac{1}{8}\pi \mu_{0}^{2}nn^{\prime }lR_{m}^{4}\sigma_{m}\frac{di}{dt}$$

Combined with Equation (6), according to Faraday’s law, the induced voltage *U*_σ_ is defined by:

(14) $$U_{\sigma }=-\frac{d\Phi_{\sigma }}{dt}=-\frac{1}{8}\pi \mu_{0}^{2}nn^{\prime }lR_{m}^{4}\sigma_{m}\frac{d^{2}i}{dt^{2}}$$

Hence, if we put the strength of the AC source (*i*(*t*) = *i*_0_ sin *ω*_0_*t*, where *i*_0_ is the amplitude of the current and *ω*_0_ represents the angular frequency of the current source) into Equation (13), the equivalent impedance *Z*_σ_ and the conductivity of the metal can be deduced as:

(15) $$Z_{\sigma }=\frac{U_{\sigma }}{i\left(t\right)}=-\frac{1}{8}\pi \mu_{0}^{2}nn^{\prime }lR_{m}^{4}\sigma_{m}\omega_{0}^{2}=\chi_{L}-\chi_{L}^{\prime }$$

(16) $$\sigma_{m}=\frac{8\left(\chi_{L}-\chi_{L}^{\prime }\right)}{\pi \mu_{0}^{2}nn^{\prime }lR_{m}^{4}\omega_{0}^{2}}$$

where the $\chi_{L}$ and $\chi_{L}^{\prime }$ refer to the impedance of the initial inductance and the inductance filled with the metal rod.

Therefore, the conductivity can be evaluated through Equation (16) by applying the parameter and impedance change in the inductive sensor.

### 2.3. Measurement Method of Inductance Based on the RLC Resonant Circuit

In order to acquire the impedance of the single-layer solenoid, the RLC circuit is engaged to analyze the proposed sensor to extract the properties of the inductance. It is known that the amplitude of the eddy current can be significantly influenced by the non-contact lift-off variation (the distance between the sensing probe and the testing metal) [30]. To avoid the disturbance induced by the lift-off effect, all the parameters are measured when the metal rod is completely inserted into the solenoid.

**Figure 2:**
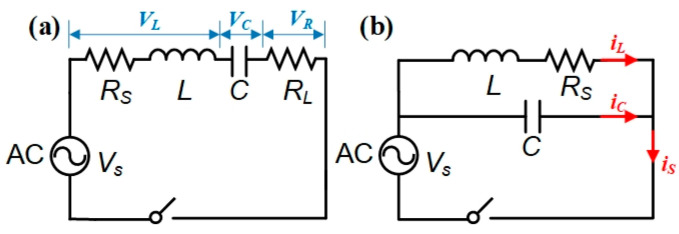
(**a**) Measurement system diagram based on the RLC circuit with excitation source in series mode and (**b**) parallel mode.

The circuit diagram of the series RLC circuit is given in Figure 2a, setting with a load resistance *R_L_* of 10 Ω, a capacitance *C* of 1 µF, and an initial inductance *L*_0_ of 1.79 mH (calculated by Equation (4), with the *N* of 480 turns, *l_c_* of 60 mm, and *R_c_* of 12 mm). The amplitude of the AC voltage source (*V_S_*) is fixed at 3 V. Applying Kirchhoff’s law, the current in each component is the same in a series RLC circuit, and we can obtain:

(17) $$\frac{V_{R}}{R_{L}}=\frac{V_{C}}{\frac{1}{j\omega C}}=\frac{V_{L}}{j\omega L+R_{S}}$$

where the *V_R_*, *V_L_*, and *V_C_* represent voltage across the load resistor, the inductor, and the capacitor, respectively. *R*_S_ denotes the resistance of the solenoid coil, which can be treated as zero for an ideal inductor, and *ω* is the angular frequency of the RLC circuit. The total impedance of the series RLC ($Z_{s}$) circuit is given by:

(18) $$Z_{s}=R_{L}+R_{S}+\chi_{C}+\chi_{L}=R_{L}+R_{S}+\;j\omega L+\frac{1}{j\omega C}$$

where *χ_C_* refers to the impedance of the capacitor.

The typical value of frequency in a series RLC circuit, for which the image part of the total impedance is zero, is named as the resonance frequency *f_r_* (corresponding to the resonant angular frequency *ω_r_*), which is defined by [13]:

(19) $$f_{r}=\frac{\omega_{r}}{2\pi }=\frac{1}{2\pi \sqrt{LC}}$$

At this moment, the impedance of the series RLC circuit reaches the minimum, indicating that the total current possesses the maximum value of *V_s_*/*(R_L_ + R_S_*). The voltage across the inductor possesses the same value as the voltage across the capacitor but in the opposite direction. Hence, by monitoring the voltage in each component or the total current, the inductance is deduced as:

(20) $$L=\frac{1}{4\pi^{2}{f_{r}}^{2}C}$$

Therefore, the inductance of the solenoid can be measured by supplying the resonance frequency and the capacitor in an RLC circuit. Subsequently, by substituting the experimental inductance into Equation (16), the conductivity of the metal can be evaluated.

To better evaluate the properties of the inductive sensor, the RLC circuit arranged in parallel mode is also used to measure the inductance of the single-layer solenoid, whose diagram is shown in Figure 2b. Applying Kirchhoff’s law, the total current is the sum of the branch currents in a parallel RLC circuit, and we can obtain:

(21) $$i_{s}=i_{L}+i_{C}=\frac{V_{s}}{\frac{1}{j\omega C}}+\frac{V_{s}}{j\omega L+R_{S}}$$

where the *i_s_*, *i_L_*, and *i_C_* refer to the summed current, the current in the inductor, and the current in the capacitor, respectively. The total impedance of the parallel RLC circuit ($Z_{p}$) is given by:

(22) $$\frac{1}{Z_{p}}=\frac{1}{R_{S}+\chi_{L}}+\frac{1}{\chi_{C}}=\frac{1}{R_{S}+j\omega L}+\frac{1}{\frac{1}{j\omega C}}$$

Then, the resonance frequency in parallel mode can be obtained when the total impedance reaches the maximum, which is derived by:

(23) $$f_{r}=\omega_{r}\;/\;2\pi =\frac{\sqrt{\frac{1}{LC}-{\left(\frac{R_{S}}{L}\right)}^{2}}}{2\pi }\approx \frac{1}{2\pi \sqrt{LC}}$$

As for the resonant state for the parallel RLC circuit, the total impedance reaches the maximum value of *L*/(*CR_S_*), demonstrating that the minimum value of *i*_s_ is equal to (*V_s_·CR_S_*)/*L*. That is, by monitoring the resonance frequency in parallel mode and substituting it into Equation (16), the inductance of the solenoid inductor can be obtained as well.

## 3. Experimental Results

### 3.1. The Effect of the Circuit Parameters on the Resonance Frequency

#### 3.1.1. Experimental Setup

**Figure 3:**
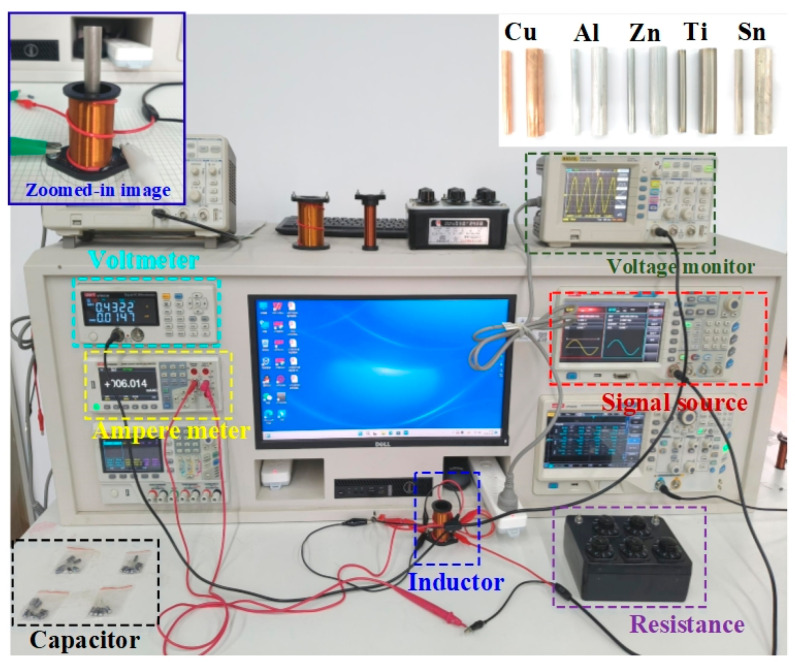
Experimental setup of the inductive sensor system. The left inset displays the zoomed-in image of the inductive sensor. The right inset shows the optical images of different metals with variable radii of 5 mm and 10 mm.

Figure 3 shows the photograph of the entire measurement system, which is composed of the electrical multi-functional testing system (including the signal sources, voltmeter, and ampere meter, purchased from Yulide Technology (Shenzhen, China) Co., Ltd., LAB—550 NeptuneLab, Dongguan, China), inductive coil (Andeli Electronics, Tianmen, China), capacitor (Kobe Semiconductor Co., Ltd., Shenzhen, China), oscilloscope (RIGOL technologies Inc., Suzhou, China, DS1052E).The inset displays the image of the testing metal with different radii, which includes the non-ferromagnetic metals, such as copper (Cu), aluminum (Al), zinc (Zn), titanium (Ti), and tin (Sn). The different metal cylinders were purchased from Huabei Technology Metal Materials, with high purity (>99.9%). The fabrication process involves pouring molten metal into a mold and then performing cylindrical cutting. To promote the testing accuracy of the electric conductivity, each type of metal cylinder has been prepared with different radii (2–10 mm) for measurement. The length of each type of metal cylinder is 10 mm, which is larger than that of the solenoid. According to Equations (5) and (20), the inductance of the solenoid can be calculated by offering the capacitance and the resonant frequency. In order to acquire the measured inductance with the highest accuracy, it is necessary to optimize the parametric factors. All the parameters utilized in the solenoid-based inductive sensor implemented with the RLC resonant circuit are summarized in Table 1. After the proper connection of the circuit, the experimental time takes several seconds to find the resonant frequency of each metal. Under the condition of keeping other parameters unchanged, the influence of the inductive sensor response characteristics with a specific element can be investigated. During the experiment, it is also worth noting that the solenoid coil and the metal cylinder should be placed at the position that coincides with the corresponding circle of a certain radius on the polar-coordinate paper to ensure that the center of the metal core is aligned with the center of the coil.

#### 3.1.2. The Influence Induced by the Capacitance

**Figure 4:**
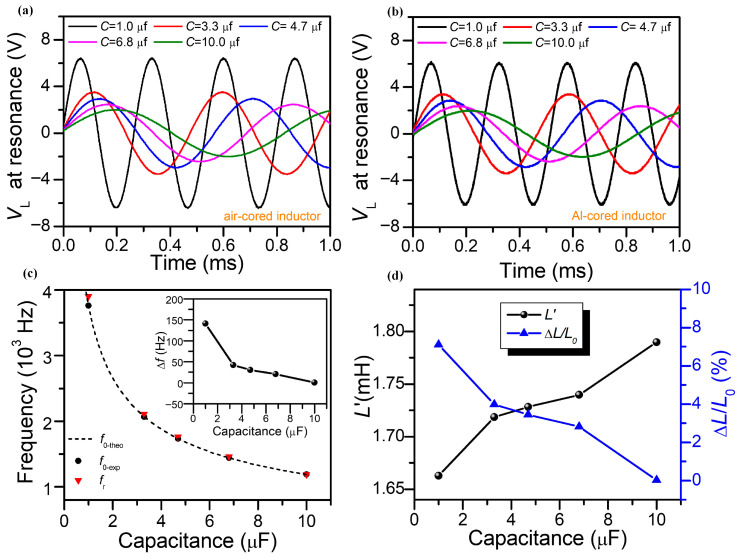
(**a**) Effect on voltages across the air-cored single-layer solenoid with different capacitances at resonance state in series mode. The black, red, blue, violet, and green lines correspond to capacitances of 1.0 μF, 3.3 μF, 4.7 μF, 6.8 μF, and 10.0 μF, respectively. (**b**) Voltages across the Al-cored single-layer solenoid at the resonant state. (**c**) Resonant frequency of the RLC circuit in series mode with the various capacitances. The dashed line represents the theoretical values of the resonant frequency as a function of the capacitance. The inset portrays the frequency change by inserting the Al rod into the single-layer solenoid. (**d**) Calculated inductance and relative change rate of the Al-cored solenoid sensor versus the capacitance.

Figure 4a depicts the voltage across the air-cored single-layer solenoid, with the capacitances of 1.0 μF, 3.3 μF, 4.7 μF, 6.8 μF, and 10.0 μF in a series RLC circuit. The *R*_C_, *N*, and *l_c_* were set to 12 mm, 480 turns, and 60 mm, respectively. It can be seen that the amplitude of *V*_L_ decreases with increasing capacitance. This result is attributed to the frequency reduction at the resonant state due to the rising capacitance, which leads to the quality factor (Q-factor) degrading as well. The Q-factor in series resonant mode (*Q_s_*) is defined as: $Q_{s}=2\pi \frac{\frac{1}{2}{{Li}_{0}}^{2}\sin^{2}\omega_{r}t+\frac{1}{2}C{\left(\frac{i_{0}}{\omega_{r}C}\cos\omega_{r}t\right)}^{2}}{{\left(\frac{i_{0}}{\sqrt{2}}\right)}^{2}(R_{L}+R_{s})\frac{2\pi }{\omega_{r}}}=\frac{\omega_{r}L}{R_{L}+R_{s}}=\frac{1}{R_{L}+R_{s}}\sqrt{\frac{L}{C}}$. However, the amplitude of *V*_L_ reduces when the single-layer solenoid is inserted by the Al core with a radius of 5 mm, as shown in Figure 4b. The different amplitudes of *V_L_* for the air-cored inductor were measured to be 6.4 V, 3.5 V, 2.9 V, 2.5 V, and 2.1 V, which correspond to capacitances of 1.0 μF, 3.3 μF, 4.7 μF, 6.8 μF, and 10.0 μF, respectively. Meanwhile, the amplitudes of *V_L_* for the Al-cored inductor were measured to be 5.9 V, 3.4 V, 2.8 V, 2.4 V, and 1.9 V, which correspond to capacitances of 1.0 μF, 3.3 μF, 4.7 μF, 6.8 μF, and 10.0 μF, respectively. Figure 4c depicts the resonant frequency of the RLC circuit in series mode with the various capacitances, where *f_0-exp_* and *f_r_* represent the measured frequency of the air-cored and Al-cored solenoid inductor. The dashed line represents the theoretical values of the resonant frequency (*f_0-theo_*) of the air-cored inductor versus the capacitance deduced by substituting Equation (5) into Equation (20), which suggests the *f_0-exp_* is consistent with the theoretical result. To better estimate the effect of *V*_L_ with different capacitance, the inset portrays the frequency change (Δ*f* = *f_0-exp_* − *f_r_*) by inserting the Al rod into the single-layer solenoid. Correspondingly, the calculated inductance of the Al-cored inductor (*L′*) versus the capacitance is portrayed in Figure 4d. The inductance of the Al-cored solenoid sensor can be obtained by providing the different resonant frequencies and capacitances. Since the sensitivity of the solenoid sensor is also affected by the pure coil inductance (*L*_0_), the normalized change rate of inductance ($\Delta L/L_{0}=(L_{0}-L^{\prime })/L_{0}$) is defined as an effective parameter to estimate the properties of the inductive sensor. It can be seen that the normalized change rate of inductance decreases with rising capacitance. The above results illustrate that the lowest capacitance of 1 μF is beneficial to increase the sensitivity of the solenoid sensor.

### 3.2. The Influence Induced by the Coil Radius

**Figure 5:**
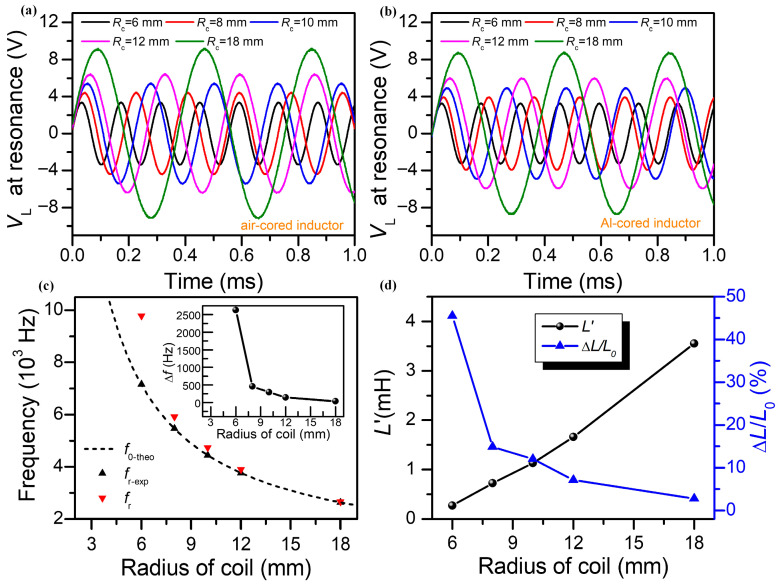
(**a**) Effect on voltages across the air-cored solenoid inductor with the coil radius of 6 mm, 8 mm, 10 mm, 12 mm, and 18 mm, respectively. (**b**) Voltages across the Al-cored solenoid inductor at the resonant state. (**c**) Resonant frequency of the RLC circuit in series mode with different coil radii. The inset shows the frequency change by inserting the Al rod into the single-layer solenoid. (**d**) Calculated inductance and relative change rate of the Al-cored solenoid sensor versus different coil radii.

Figure 5a depicts the effect on voltage across the air-cored solenoid inductor at the resonant state in series mode, with the coil radius of 6 mm, 8 mm, 10 mm, 12 mm, and 18 mm, respectively. The *C*, *N*, and *l_c_* were still set to 1 μF, 480 turns, and 60 mm, respectively. It shows that the amplitude of the *V*_L_ promotes with the rising *R_c_*, but the frequency of the *V_L_* declines. This result is ascribed to the higher inductance of the coil, leading to a higher Q-factor, but it decreases the resonant frequency. The different amplitudes of *V_L_* for the air-cored inductor were measured to be 3.3 V, 4.3 V, 5.4 V, 6.4 V, and 9.0 V, which correspond to coil radii of 6 mm, 8 mm, 10 mm, 12 mm, and 18 mm, respectively. Figure 5b portrays that the amplitude of *V*_L_ of the Al-cored solenoid also grows with the increasing *R_c_*, which is attributed to an inductive increment caused by the higher inductance of the coil. The amplitudes of *V*_L_ for the Al-cored inductor were measured to be 3.0 V, 3.8 V, 4.8 V, 5.9 V, and 8.6 V, which correspond to coil radii of 6 mm, 8 mm, 10 mm, 12 mm, and 18 mm, respectively. Nevertheless, the amplitude of *V_L_* of the Al-cored solenoid is smaller than that of the air-cored solenoid when the coil radius is the same. This phenomenon is caused by the obstructive magnetic field due to the eddy current loss in metal. Similarly, Figure 5c points out the resonant frequency of the RLC circuit in series mode with different coil radii, which indicates that the measured resonant frequency of the air-cored inductor fits well with the theoretical calculation. It is also worth noting that the *f_r_* is heavily dependent on the *R_c_*, which consequently helps the inductance change in the solenoid sensor, as proved in Figure 5d. With the *R_c_* decreasing, it is apparent that both the resonant frequency and inductance change rate show a rising tendency. However, it is improper to increase the sensitivity of the inductance by continuously reducing the radius of the coil. In our analytic solution, the circular induced eddy currents at excessively high frequencies involve taking into account the higher-order Bessel functions [12], which reduces the accuracy of the measured conductivity. Moreover, the induced electric field is related to the size of the inserted metal, and the external magnetic field is distributed throughout the depth of the conductive cylinder at a relatively low frequency to guarantee that the whole sample is probed. Indeed, it is a trade-off between the resonant frequency of the RLC circuit and the solenoid size.

Therefore, in the following experiments, *C*, *R_c_*, *N*, and *l_c_* were set to 1 μF, 12 mm, 480 turns, and 60 mm, respectively. These parameters can not only ensure the sensitivity of the inductance change but also minimize the higher-order effect of eddy current at high frequency and allow more variations in the size of the metal.

### 3.3. Inductive Response Characteristics of Different Testing Modes

**Figure 6:**
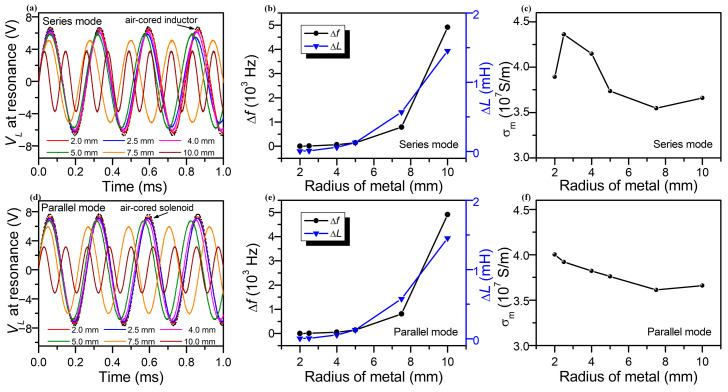
(**a**) Voltage across the Al-cored solenoid inductor in series mode at resonant state, with the core radius of 2.0 mm, 2.5 mm, 4.0 mm, 5.0 mm, 7.5 mm, and 1.0 mm. The black dashed line represents the voltage across the air-cored solenoid inductor. (**b**) The change in signal frequency and calculated inductance induced by the Al core with variable radius in series mode. (**c**) Measurement of the conductivity of the Al as a function of the core radius in series mode. (**d**) Voltage across the Al-cored solenoid inductor in parallel mode at the resonant state. (**e**) The change in signal frequency and calculated inductance induced by the Al core with variable radius in parallel mode. (**f**) Measurement of the conductivity of the Al as a function of the core radius in parallel mode.

To confirm the effect of different testing modes, the inductive response characteristics of the Al-cored solenoid sensor were measured in both the series resonant mode and parallel resonant mode. Figure 6a draws the voltage across the solenoid inductor with the Al-cored radii of 2.0 mm, 2.5 mm, 4.0 mm, 5.0 mm, 7.5 mm, and 1.0 mm, respectively, which is tested in a series RLC circuit at the resonant state. The fill factor increases with the rising radius of the metal cylinder. The amplitude of *V_L_* decreases with the increasing radii and shows a lower value than that of the air-cored solenoid (which is depicted in the black dashed line). The amplitude of *V_L_* for the air-cored inductor in series mode was measured to be 6.7 V, while the *V_L_* for the Al-cored inductor was measured to be 6.5 V, 6.3 V, 6.1 V, 5.8 V, 5.1 V, and 3.6 V, which correspond to metal radii of 2.0 mm, 2.5 mm, 4.0 mm, 5.0 mm, 7.5 mm, and 10 mm, respectively. Moreover, the increment of the frequency and the inductance shows the rising trend with the metal radius as well, as proved in Figure 6b. Based on Equations (16) and (23), the experimental conductivities of different Al cylinders were calculated, as given in Figure 6c. The testing conductivity of Al measured by the series RLC circuit was estimated to be 3.89 × 10^7^ S/m, 4.37 × 10^7^ S/m, 4.14 × 10^7^ S/m, 3.72 × 10^7^ S/m, 3.55 × 10^7^ S/m, and 3.65 × 10^7^ S/m, corresponding to radii of the metal of 2.0 mm, 2.5 mm, 4.0 mm, 5.0 mm, 7.5 mm, and 1.0 mm, respectively. Analogously, the voltage across the Al-cored solenoid inductor shows a similar tendency with the various core radii in parallel mode at the resonant state, as given in Figure 6d. The amplitude of *V_L_* for the air-cored inductor in parallel mode was measured to be 7.6 V, while the *V_L_* for the Al-cored inductor with a metal radius of 2.0 mm was measured to be 6.5 V. The inductance changes can also be obtained from the resonance frequency of the parallel RLC mode, as shown in Figure 6e. Due to the effect of the eddy current in non-ferromagnetic metal, the inductance changes were 1.7 × 10^−3^ mH, 7.2 × 10^−3^ mH, 4.9 × 10^−2^ mH, 0.13 mH, 0.57 mH, and 1.45 mH, which correspond to metal radii of 2.0 mm, 2.5 mm, 4.0 mm, 5.0 mm, 7.5 mm, and 1.0 mm, respectively. As shown in Figure 6f, the result determines that the conductivity of the Al shows a falling tendency from 4.01 × 10^7^ S/m to 3.65 × 10^7^ with the increasing metal radii. It is apparent that the relation between the inductance and conductivity evaluated by the resonance frequency shows an analogous response for both series RLC circuits and parallel RLC circuits.

## 4. Discussion

### 4.1. Conductivity Measurement Based on the Single-Layer Solenoid Sensor

**Figure 7:**
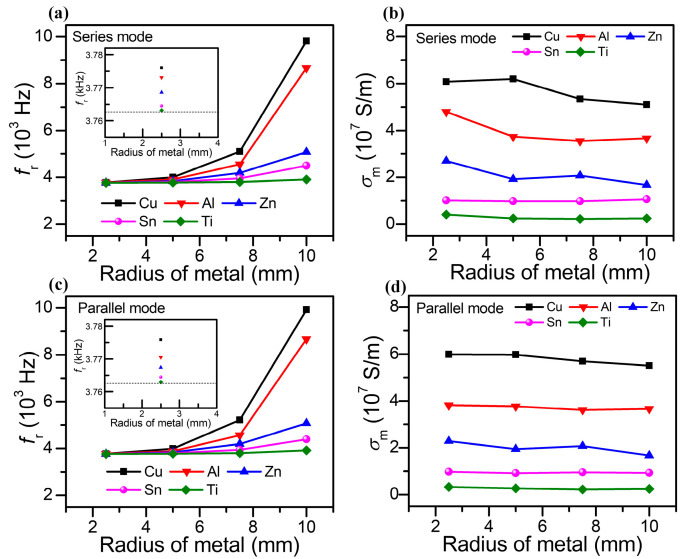
(**a**) Resonant frequency of the RLC circuit versus the metal radii of different metal-cored inductors in series mode. The inset is the zoomed-in frequency at the resonant state with the metal radius of 2.5 mm. (**b**) Calculated conductivities of various metals with different radii extracted from the parameters in the series circuit. (**c**) Resonant frequency of the RLC circuit versus the metal radii of different metal-cored inductors in parallel mode. (**d**) Calculated conductivities of various metals with different radii extracted from the parameters in a parallel circuit.

To further analyze the inductive sensor performance, Figure 7a depicts the resonant frequency of the RLC circuit versus the metal radii of different metal-cored inductors in series mode. The result shows that the non-ferromagnetic metals (such as Cu, Al, Zn, Sn, and Ti) exhibit a positive influence on the *f_r_*. Since the inductance of the single-layer solenoid is inversely proportional to the square of the resonance frequency, the inductance increment of non-ferromagnetic metal can change into different degrees of negative increment, which originates from various eddy current losses due to the conductivity of different metals. This phenomenon is consistent with our theoretical analysis. To express more details of the *f_r_,* the inset gives the zoomed-in frequency at the resonant state with the metal radius of 2.5 mm, which satisfies the above-mentioned variation trend. The conductivities of the various metals with different radii extracted from the parameters in the series circuit are determined based on Equation (16), as given in Figure 7b. It turns out that the conductivity exhibits different values with respect to the metal radius measured in the series RLC mode. The resonant frequency of the RLC circuit versus the metal radii of different metal-cored inductors in parallel mode is represented in Figure 7c. The *f_r_* of the non-ferromagnetic metal enhances with the increasing *R_m_* as well. However, the calculated *σ*_m_ measured by parallel mode possesses better stability than that of the series mode, as illustrated in Figure 7d. This result can be ascribed to the variant resonant response of different modes. The Q-factor in parallel resonant mode (*Q_p_*) is defined as: $Q_{p}=2\pi \frac{\frac{1}{2}{{Li}_{L0}}^{2}}{{\left(\frac{i_{L0}}{\sqrt{2}}\right)}^{2}R_{s}\frac{2\pi }{\omega_{r}}}=\frac{L\omega_{r}}{R_{s}}=\frac{L}{R_{s}}\sqrt{\frac{1}{LC}-{\left(\frac{R_{S}}{L}\right)}^{2}}=\frac{1}{R_{s}}\sqrt{\frac{L}{C}-{R_{s}}^{2}}$, where *i_L_0__* is the amplitude of the current in the inductor. The condition for making *Q_p_* greater than *Qs* is defined by: $R_{L}>R_{s}(\frac{1}{\sqrt{1-{\left(\frac{R_{s}}{\sqrt{L\;/\;C}}\right)}^{2}}}-1)$*. R_S_* can always be treated as zero for an ideal inductor; however, this resistance exists in the experiment. The resistance of the proposed solenoid coil (~2.7 Ω, measured by digital multimeter which is included in the multi-functional testing system) is lower than the *R_L_* of 10 Ω, which leads to a higher Q-factor in parallel mode than that in series mode. This result makes the acquisition of resonant frequency more precise in parallel mode, which further enhances the accuracy and stability for conductivity measurement.

Particularly, our approach is valid for driving frequencies up to several kilohertz. Once the frequency keeps rising, the higher-order items of the analytic solution for eddy current should be considered, which account for the deviation of the experimental result. Hence, this range is sufficient to perform a relatively precise measurement of the conductivity of metal cylinders, and the skin effect [31] can be ignored as well.

### 4.2. Simulated Analysis of the Solenoid Sensor

**Figure 8:**
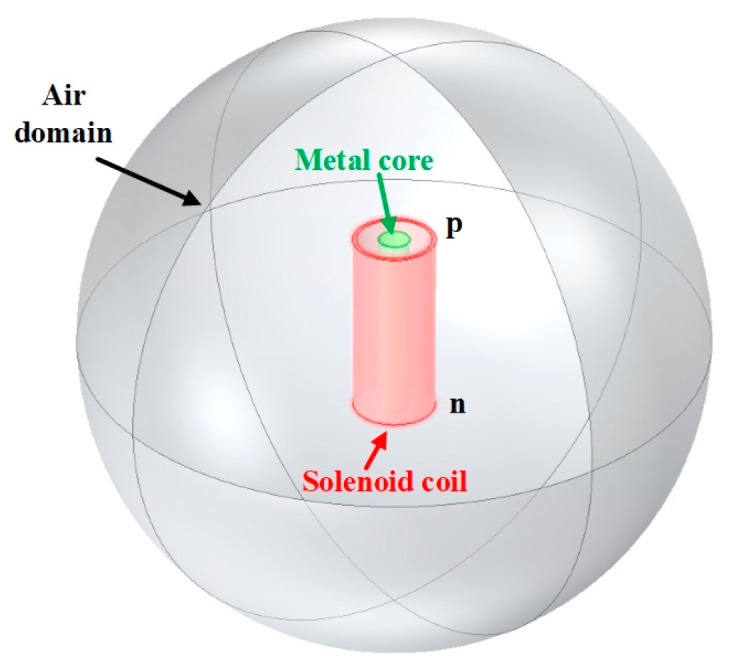
The geometry of the 3D FEM model.

Compared with the analytical method, Figure 8 portrays the three-dimensional (3D) simulation model of the solenoid sensor supplemented to the RLC equivalent circuit representation by employing Comsol Multiphysics, which is implemented using the finite-element method (FEM). The material of the coil is copper, which is wrapped in the spherical air with the same parameter settings as in Section 2.1. The virtual equivalent circuit is constructed by the AC/DC module from point “p” to “n”, with the coil being connected in both the series mode and parallel mode. The simulation result for the inductive sensor is excited by the same condition as in Section 2.3, and the voltage output of the single-layer solenoid is expressed by the “external U versus I” module, which can obtain the inductive behavior and the field distribution in the media. It is worth noting that all the media are only described by the linear electromagnetic field model, which is proper for non-ferromagnetic metal.

**Figure 9:**
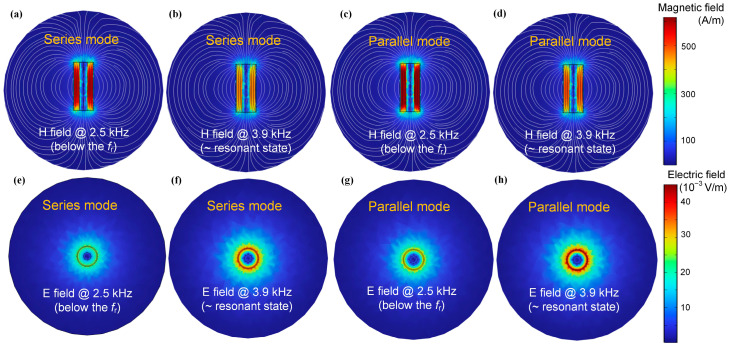
(**a**) Simulated distribution of magnetic field ***H*** for the Al-cored single solenoid sensor at 2.5 kHz and (**b**) 3.9 kHz in series mode. (**c**) Simulated distribution of magnetic field ***H*** for the Al-cored single solenoid sensor at 2.5 kHz and (**d**) 3.9 kHz in parallel mode. (**e**) Electric field ***E*** distribution of the Al-cored single-solenoid sensor at 2.5 kHz and (**f**) 3.9 kHz in series mode. (**g**) Electric field ***E*** distribution of the Al-cored single-solenoid sensor at 2.5 kHz and (**h**) 3.9 kHz in parallel mode.

To confirm the physical mechanism of the inductive characteristics of the solenoid sensor, the simulated intensity distribution of the eddy current is expressed by Comsol Multiphysics, with application of the FEM [32]. Since the magnetic field ***H*** can be used to represent the inductive mode of the metal-cored single-layer solenoid, the magnetic field distribution of the Al-cored inductive sensor with a metal radius of 5 mm at 2.5 kHz (below the resonance frequency) and 3.9 kHz (around the resonant frequency) in series mode is shown in Figure 9a,b, by setting the conductivity of Al to 3.77 × 10^7^ S/m [33]. The result demonstrates that the magnetic field of the air region within the solenoid below the *f_r_* is significantly lower compared to the magnetic field around the *f_r_*, resulting from the eddy current inside the metal rod, which can obtain the maximum at resonance which leading to a decrease in the magnetic field of the air region within the solenoid. Similarly, Figure 9c,d depict the magnetic field distribution at 2.5 kHz and 3.9 kHz in the parallel mode, suggesting a similar trend as the series mode. However, it can be seen that the ***H*** distribution in parallel resonance is slightly different from the ***H*** distribution in series resonance, which is ascribed to the impedance variation in different modes. Figure 9e,f show the corresponding electric field ***E*** distributions around the inductive solenoid in series mode, simulated at 2.5 kHz and 3.9 kHz, while the ***E*** distribution of the sensor in parallel mode is given in Figure 9g,h. The strength of the electric field in series resonance is lower than that in parallel mode, which is attributed to the smaller voltage across the inductor at series resonance. These simulation results can also verify the theoretical analysis in Section 2.

### 4.3. Relative Standard Deviation and the Relative Error of Conductivity Measurement

To estimate the sensing accuracy of the inductor, the relative standard deviation (RSD) and the relative error (RE) of the conductivity are calculated as shown in Table 2, which are defined as:

(24) $$RSD=\frac{1}{\bar{x}}\sqrt{\frac{\sum_{i=1}^{n}{\left(x_{i}-\bar{x}\right)}^{2}}{n-1}}\times 100\%$$

(25) $$RE=\frac{\bar{\sigma_{m}}-\sigma_{r}}{\sigma_{r}}\times 100\%$$

where *n* is the measurement time, $\bar{x}$ is the average value, and $x_{i}$ is the result of the *i*th measurement. Meanwhile, $\bar{\sigma_{m}}$ denotes the average value of the conductivity of the metal, and $\sigma_{r}$ refers to the reference result of the conductivity implemented by the four-point probe measurement (Four-pin Technology, ST2258C, Guangzhou, China). It turns out that the maximum RSD and RE of the conductivity are 10.55% for Zn and 13.04% for Ti operated by the series resonant mode. Despite this result, it still fits well with the reference value, which possesses an accuracy of 86.95%. The errors of the conductivity measurement can be attributed to the coil position offset, coil resistance, and surface roughness of metal cylinders, etc., which can be mitigated by reducing the coil resistance and adding the platform of displacement alignment. From Table 2, it is apparent that the stability and accuracy of the inductive sensor measured by parallel mode are higher than that of series mode, which can be attributed to the higher sensitivity of the solenoid sensor operated at parallel resonance. This validates our accuracy of measurement based on a single-layer solenoid sensor and certifies it to be an effective approach in determining the electrical conductivity of different metals.

## 5. Conclusions

Above all, we proposed a novel non-contact way to measure the conductivity of the non-ferromagnetic metals with the single-layer solenoid sensor. Through experiments performed on an RLC resonance circuit, the optimal measurement condition can be obtained by changing the capacitance and the coil size. By comparing the inductance change between the air-cored and metal-cored solenoid, the conductivities of different metals can be deduced in both the series mode and parallel mode, with the application of the eddy current considering the edge effect. Furthermore, the simulation of the inductive model was also engaged to express the distribution of the magnetic field of the solenoid sensor, which is consistent with the experimental measurement. At the same time, conductivity measurement with a higher stability and accuracy was obtained by employing the parallel resonance mode, which is attributed to the more precise acquisition of resonant frequency in parallel mode. The calculated conductivities of the non-ferromagnetic metals are all in good agreement with the reference value, with the highest relative error of 8.69% and maximum relative standard deviation of 8.03% in parallel mode. These results attest that the single-layer solenoid sensor can feasibly provide an approach for conductivity measurement in non-ferromagnetic metals with a cylindrical shape. Our work indicates that the single-layer solenoid can be utilized to determine the electric conductivity and is expected to offer a theoretical foundation for the inductive sensor.

## Figures and Tables

**Table 1:** The parameters of the solenoid-based inductive sensor implemented with the RLC resonant circuit.

Parameter	Value	Description
*N*	480	Number of turns in the coil
*l_c_* (mm)	60	Length of the coil
*R_c_* (mm)	6, 8, 10, 12, 18	Radius of the coil
*l_m_* (cm)	10	Length of the metal rod
*R_m_* (mm)	2–10	Radius of the metal rod
*R_S_* (Ω)	~0	Resistance of the coil
*R_L_* (Ω)	10	Load resistance
*C* (µF)	1.0, 3.3, 4.7, 6.8, 10	Capacitance
*L* (mH)	1/$\left(4\pi^{2}f_{r}^{2}C\right)$	Inductance of the solenoid
*V_S_* (V)	3	Amplitude of the voltage source
*f_r_* (Hz)	1/$\left(2\pi \sqrt{LC}\right)$	Resonant frequency
*ω_r_* (Hz)	1/$\sqrt{LC}$	Resonant angular frequency

**Table 2:** Comparison of the conductivity measurement based on the solenoid sensor with the four-point probe method.

Metal	Reference Data	Proposed Data					
		In Series Mode			In Parallel Mode		
	$\sigma_{r}$ **(10^7^ S/m)**	**Measured** $\bar{\sigma_{m}}$ **(10^7^ S/m)**	**RSD** **(%)**	**RE** **(%)**	**Measured** $\bar{\sigma_{m}}$ **(10^7^ S/m)**	**RSD** **(%)**	**RE** **(%)**
**Cu**	5.86	5.68	9.51	−3.07	5.79	5.92	−1.20
**Al**	3.79	3.85	7.38	1.58	3.65	2.76	−3.69
**Zn**	1.71	1.88	10.55	9.94	1.84	6.39	7.60
**Sn**	0.89	0.96	1.86	7.86	0.93	1.40	4.49
**Ti**	0.23	0.26	5.90	13.04	0.25	8.03	8.69

## Data Availability

Data are contained within the article.
